# Supplementary material for: Multi‐dataset identification of innovative feature genes and molecular mechanisms in keratoconus
Source: J Cell Mol Med. 2024 Sep 19;28(18):e70079. doi: 10.1111/jcmm.70079 (PMC11412914; doi:10.1111/jcmm.70079)
Supplement: Supplementary file 2 — Material S2. [file JCMM-28-e70079-s002.zip › 7.GSEA analysis/2.ATOH7/KEGG_ALANINE_ASPARTATE_AND_GLUTAMATE_METABOLISM.html]

Details for gene set KEGG\_ALANINE\_ASPARTATE\_AND\_GLUTAMATE\_METABOLISM[GSEA]

|  || Dataset | ATOH7.group.cls#h\_versus\_l |
| Phenotype | group.cls#h\_versus\_l |
| Upregulated in class | h |
| GeneSet | KEGG\_ALANINE\_ASPARTATE\_AND\_GLUTAMATE\_METABOLISM |
| Enrichment Score (ES) | 0.41146913 |
| Normalized Enrichment Score (NES) | 1.3754202 |
| Nominal p-value | 0.06412826 |
| FDR q-value | 0.33452457 |
| FWER p-Value | 0.922 |
Table: GSEA Results Summary

  

Fig 1: Enrichment plot: KEGG\_ALANINE\_ASPARTATE\_AND\_GLUTAMATE\_METABOLISM      
 Profile of the Running ES Score & Positions of GeneSet Members on the Rank Ordered List

  

| SYMBOL | TITLE | RANK IN GENE LIST | RANK METRIC SCORE | RUNNING ES | CORE ENRICHMENT || 1 | ACY3 | na | 308 | 0.622 | 0.1410 | Yes |
| 2 | ASPA | na | 674 | 0.503 | 0.2518 | Yes |
| 3 | ALDH5A1 | na | 1652 | 0.373 | 0.3135 | Yes |
| 4 | AGXT | na | 3715 | 0.248 | 0.3138 | Yes |
| 5 | GAD1 | na | 4303 | 0.225 | 0.3512 | Yes |
| 6 | CPS1 | na | 5543 | 0.184 | 0.3599 | Yes |
| 7 | AGXT2 | na | 6514 | 0.157 | 0.3697 | Yes |
| 8 | GLS2 | na | 7210 | 0.138 | 0.3829 | Yes |
| 9 | GFPT2 | na | 7391 | 0.133 | 0.4098 | Yes |
| 10 | CAD | na | 8279 | 0.113 | 0.4115 | Yes |
| 11 | GFPT1 | na | 9726 | 0.086 | 0.3905 | No |
| 12 | GPT2 | na | 10846 | 0.068 | 0.3747 | No |
| 13 | ASS1 | na | 10957 | 0.067 | 0.3876 | No |
| 14 | IL4I1 | na | 11846 | 0.055 | 0.3752 | No |
| 15 | ALDH4A1 | na | 12888 | 0.042 | 0.3552 | No |
| 16 | GLUD2 | na | 13502 | 0.035 | 0.3461 | No |
| 17 | ASNS | na | 13578 | 0.035 | 0.3523 | No |
| 18 | GLS | na | 16616 | 0.008 | 0.2666 | No |
| 19 | GLUL | na | 16859 | 0.006 | 0.2611 | No |
| 20 | PPAT | na | 17099 | 0.004 | 0.2550 | No |
| 21 | GOT1 | na | 17581 | -0.000 | 0.2413 | No |
| 22 | ADSL | na | 18584 | -0.009 | 0.2146 | No |
| 23 | NIT2 | na | 22001 | -0.043 | 0.1265 | No |
| 24 | GLUD1 | na | 22595 | -0.050 | 0.1214 | No |
| 25 | GOT2 | na | 23507 | -0.062 | 0.1101 | No |
| 26 | ABAT | na | 23756 | -0.066 | 0.1189 | No |
| 27 | ASL | na | 24987 | -0.085 | 0.1040 | No |
| 28 | GPT | na | 29820 | -0.205 | 0.0141 | No |
| 29 | GAD2 | na | 31008 | -0.247 | 0.0396 | No |
| 30 | DDO | na | 31742 | -0.280 | 0.0861 | No |
Table: GSEA details [plain text format]

  

Fig 2: KEGG\_ALANINE\_ASPARTATE\_AND\_GLUTAMATE\_METABOLISM      
 Blue-Pink O' Gram in the Space of the Analyzed GeneSet

  

Fig 3: KEGG\_ALANINE\_ASPARTATE\_AND\_GLUTAMATE\_METABOLISM: Random ES distribution      
 Gene set null distribution of ES for **KEGG\_ALANINE\_ASPARTATE\_AND\_GLUTAMATE\_METABOLISM**
